# Correction: Inhibitory effect of a genistein derivative on pigmentation of Guinea pig skin

**DOI:** 10.1039/c8ra90012d

**Published:** 2018-02-13

**Authors:** Quancheng Zhou, Chuanxing Feng, Zheng Ruan

**Affiliations:** State Key Laboratory of Food Science and Technology, School of Food Science and Technology, Nanchang University Nanchang 330047 China ruanzheng@ncu.edu.cn +86-791-88304402; School of Agricultural Engineering and Food Science, Shandong University of Technology Zibo 255049 China

## Abstract

Correction for ‘Inhibitory effect of a genistein derivative on pigmentation of Guinea pig skin’ by Quancheng Zhou *et al., RSC Adv.*, 2017, **7**, 7914–7919.

Affiliation a as published was incorrect; the correct affiliation is given below.

The Royal Society of Chemistry apologises for these errors and any consequent inconvenience to authors and readers.

## Supplementary Material

